# Effect of Co-Application of *Azospirillum brasilense* and *Rhizobium pisi* on Wheat Performance and Soil Nutrient Status under Deficit and Partial Root Drying Stress

**DOI:** 10.3390/plants12173141

**Published:** 2023-08-31

**Authors:** Bushra Ahmed Alhammad, Muhammad Saqlain Zaheer, Hafiz Haider Ali, Akhtar Hameed, Kholoud Z. Ghanem, Mahmoud F. Seleiman

**Affiliations:** 1 Biology Department, College of Science and Humanity Studies, Prince Sattam Bin Abdulaziz University, P.O. Box 292, Riyadh 11942, Saudi Arabia; b.alhammad@psau.edu.sa; 2 Department of Agricultural Engineering, Khwaja Fareed University of Engineering and Information Technology, Rahim Yar Khan 64200, Pakistan; 3 Department of Agriculture, Government College University, Lahore 54000, Pakistan; dr.haiderali@gcu.edu.pk; 4 Institute of Plant Protection, MNS University of Agriculture Multan, Multan 61000, Pakistan; akhtar.hameed@mnsuam.edu.pk; 5 Department of Biological Science, College of Science and Humanities, Shaqra University, Riyadh 11961, Saudi Arabia; kghanem@su.edu.sa; 6 Plant Production Department, College of Food and Agriculture Sciences, King Saud University, P.O. Box 2460, Riyadh 11451, Saudi Arabia; 7 Department of Crop Sciences, Faculty of Agriculture, Menoufia University, Shibin El-Kom 32514, Egypt

**Keywords:** crop yield, water stress, partial root drying, rhizobacteria, soil fertility

## Abstract

Water management techniques are improving at the farm level, but they are not enough to deal with the limited availability of water and increased crop yields. Soil microbes play a vital role in nitrogen fixation, improving soil fertility and enhancing plant growth hormones under drought conditions. Therefore, this study was conducted to investigate the impact of water management combined with *Azospirillum brasilense* and *Rhizobium pisi* on wheat crop productivity and soil properties in dry regions. Three water management techniques were compared, normal irrigation as a control (C), deficit irrigation (DI), and partial root drying irrigation (PRD), together with the interaction of plant-growth-promoting rhizobacteria (PGPR). Experiments were conducted with six treatments in total: T_1_ = C + No PGPR, T_2_ = C + PGPR, T_3_ = DI + No PGPR, T_4_ = DI + PGPR, T_5_ = PRD + No PGPR, and T_6_ = PRD + PGPR. The highest grain yield was achieved in the control irrigation treatment using seeds inoculated with rhizobacteria, followed by control treatment without any inoculation, and the lowest was recorded with deficit irrigation without rhizobacteria inoculated in the seeds. However, PRD irrigation resulted in significantly higher plant growth and grain yield than the DI treatment. PGPR inoculation combined with PRD resulted in a 22% and 20% higher number of grains per spike, a 19% and 21% higher grain yield, and a 25% and 22% higher crop growth rate compared to rhizobacteria inoculation combined with the DI system in 2021-22 and 2022-23, respectively. This increase was due to the higher production of growth hormones and higher leaf area index under water-limited conditions. A greater leaf area index leads to a higher chlorophyll content and higher food production for plant growth.

## 1. Introduction

Wheat (*Triticum aestivum* L.) was the first domesticated cereal and is the most important staple food worldwide. It is the most widely grown crop and fulfills the nutrient needs of more than 40% of the world’s population [1]. Wheat is a cereal crop and is severely affected by seasonal drought. It is mostly grown in the arid and semi-arid regions of the country [2]. Poor water management and the unavailability of water are the main cause of low yields in these areas. Growth parameters are affected by poor metabolism and physiological functions when crops face dry periods [3]. The reduction in crop yield due to water stress varies according to the intensity and timing of drought stress [4]. Wheat is not only the staple crop but the most important crop for the country’s economy. Its production in arid and semi-arid regions is highly restricted due to the poor availability of water and low rainfall [5]. Limited water availability and seasonal drought have intense effects on the wheat crop yield, so it is very difficult to attain the actual potential [6]. Water management techniques are improving at the farm level, but they are not enough to counter the effects of limited water availability and increase crop yield [7,8]. The cropping area and wheat crop yield will decrease day by day; on the other hand, soil salinity is increasing in arid regions [5]. Inefficient irrigation systems in arid regions are the main reason for the increasing salinity and poor plant growth. The unavailability of water is causing a decrease in the wheat cropping area under arid conditions [9]. The unreliability and inequity of rainfall and the seasonal drought conditions are also key reasons for decreasing crop yields. In general, wheat is sown on flat land and flood irrigation is applied, leading to a significant loss of water. The sowing of wheat on a bed or furrow is also useful to save water, but still not as effective under limited water availability [10].

Using a suitable irrigation method in dry regions is the main priority for farmers to obtain maximum crop yields with the available water. Water management and different agronomic practices are highly effective in increasing crop yields with the optimal utilization of the available resources, especially in dry regions [11]. The flood irrigation method is the most widely used practice for farmers, as it requires less labor and is low-cost, but farmers are now moving towards the sowing of wheat crops on beds and furrows to save irrigation water. Water management techniques in dry areas can directly affect crop yield [12]. Water deficit irrigation (DI) is an irrigation method in which less water is applied than the crop requires, but drought conditions do not exist at any growth stage. This method is effective under very low water availability, and flood irrigation leads to a loss of water [13]. Partial root-zone drying irrigation (PRDI) is another water management technique in which irrigation is first applied on one side of the roots while the other side remains dry, and the next time is applied on the other side of the roots. A water saving of 50% has been reported from the use of DI and PRDI, but Iqbal et al. [14] have recommended PRDI as more suitable irrigation method to obtain higher crop yields. It was also reported that during PRDI, plants modify their internal metabolic and physiological functions and try to close their stomata due to internal plant signaling, as irrigation water is limited or unavailable, but water is available in the soil on one side of the roots and plants can continue their metabolic functions [15].

Low soil fertility and drought are the leading threats to wheat production. The continual use of inorganic chemical fertilizers can lower soil productivity. Micro-organisms are very beneficial to crop production and nutrient availability in the soil [16]. Plant-growth-promoting rhizobacteria (PGPR) are a group of microbes that play a vital role in nitrogen fixation, improving soil fertility, enhancing nutrient uptake by plants, and increasing the amounts of growth hormones in the plants, helping to improve crop yield. PGPR also improve the tolerance of plants to water stress and pests [17]. These bacteria are naturally present in the soil and colonize the root zone, and some bacteria form nodules in leguminous crops that improve plant growth. PGPR also improve soil fertility by enhancing nutrient contents and breaking down complex nutrients into a simple form available for plant uptake [18]. Inoculation of crop seeds with PGPR during sowing improves crop biomass and production [19]. PGPR fix atmospheric nitrogen and increase soil fertility. They also increase the resistance of plants to oxidative and drought stress with the production of water-soluble vitamins, riboflavin, pantothenic acid, thiamine, cytokinins, and many other growth-promoting hormones [20]. Siddhartha et al. [21] and Cohen et al. [22] reported root enhancement and more water content in plants with the application of *Azospirillum brasilense*, while higher nutrient supply and growth hormone production was reported from *Rhizobium pisi* [23,24]. Yan et al. [25] and Nie et al. [26] reported that the different environmental conditions can affect the soil fertility and growth of different plants. Water availability and its quality can damage the whole plant physiological mechanism [27,28]. Soil fertility and soil structure also affect plant growth [4,29]. Zhang et al. [30] reported that the availability of beneficial soil microbes can produce the antibiotics to improve plant growth and root biomass. An increase in root biomass can increase nitrogen uptake [31]. Both these bacteria were also reported by many researchers to helpful for plant growth promotion under water stress condition [23,24,32,33,34], but nothing is clear about their combined application under drought stress.

Inoculation of crop seeds with PGPR is an effective method to enhance long-term soil productivity and obtain a higher crop yield [35,36]. Ecofriendly crop production is also possible with the maximum utilization of soil microbes in agricultural crop production. Different bacterial groups, such as Bacillus, Azospirillum, Enterobacter, Pseudomonas, and Agrobacterium, are used in seed inoculation [37,38], which is reported to be very beneficial for soil productivity, enhancing crop yields and also improving drought stress tolerance in wheat [39]. The interaction of PGPR with PRD and DI irrigation systems in wheat crop production is not investigated enough, and therefore there is a need to understand the soil properties and plant growth/yield improvement [40]. PGPR might modify plant growth under DI or PRDI systems, but investigations on such are limited.

Therefore, this study aimed to investigate the physiological effects of PRD, DI, and FI systems combined with the PGPR inoculation of wheat seeds under field and wirehouse conditions. Further, the current study was also designed to test the hypothesis that combining PGPR with a suitable irrigation method can improve plant growth and soil properties under limited water conditions.

## 2. Materials and Methods

### 2.1. Plant Material and Experimental Unit

This experiment was conducted at greenhouse, during 2021–2022 and 2022–2023, with a complete randomized design (CRD) to investigate the impact of water management combined with microbial inoculation on crop productivity and soil properties. The sowing date for seeds was 5th November. Three water management practices were used in the experiment: normal irrigation as a control (C), deficit irrigation (DI), and partial root drying (PRD) irrigation, all with and without the use of plant-growth-promoting rhizobacteria (PGPR). The experiments consisted of six treatments, each having three replicates: T_1_ = Control + No PGPR, T_2_ = C + PGPR, T_3_ = DI + No PGPR, T_4_ = DI + PGPR, T_5_ = PRD + No PGPR, T_6_ = PRD + PGPR. PGPR was applied as a combination of two bacterial strains, *Azospirillum brasilense* and *Rhizobium pisi*. The wheat variety Galaxy-2013 was used in the current investigation. The pot size was 2 × 2 × 2 feet with 20 kg of soil per pot, and soil analysis was performed before the start of the experiment. The mean annual temperature at the experimental site was 27 °C and the average humidity was 55%. PGPR strains of *Azospirillum brasilense* and *Rhizobium pisi* were obtained from the Government College University, Lahore. Irrigation was applied on critical growth stages (Tillering (30 days after sowing (DAS)), booting (60 DAS), anthesis (90 DAS), and grain filling stage (110 DAS)) of the wheat crop when the crop required the irrigation water. The timing of the irrigation for PRD was also the same as for the control treatment. Recommended fertilizers were applied equally to all the experimental units.

Seeds were sown with seed inoculation according to the procedure described by Fukami et al. [39]. Ten pre-germinated wheat seeds were sown in each pot. After eight days of emergence, the seedlings of the PRD treatments were uprooted, and the roots were divided in half. Wheat seedlings were repotted into special pots with partitions that allowed the roots of each plant to be separated equally between each portion. Partitioned wheat seedlings were watered for 6 days. One side was irrigated on one event, and the other side was irrigated on the next event. Deficit irrigation (DI) was applied so that the seedlings received 60% of the amount of water applied in the control treatment. Loam soil was used in the experiment and the properties of the pot are given in Table 1.

### 2.2. Plant Analysis: Morpho-Biochemical Traits

Plant height, spike length, the number of spikelets per spike and grains per spike, the 1000-grain weight, and yield per plant were recorded when the crop matured using standard procedures (Bhutta et al. [41]). The leaf area index (LAI), crop growth rate (CGR), and relative growth rate (RGR) were calculated according to the procedure described by Gardner et al. [42]. Leaf samples of 0.5 g were placed in distilled water for 24 h at 4 °C to determine the turgid weight, and after that the samples were oven-dried at 65 °C for 48 h to obtain the dry weight (g). The relative water content (RWC) was determined using the formula reported by Barr and Weatherley [43]:

RWC (%) = [(FW − DW)/(TW − DW)] × 100(1)

Stomatal conductance and the photosynthesis rate were measured using an infrared gas analyzer (Cl-340 Handheld Photosynthesis System, Camas, WA, USA), and a chlorophyll meter (CL-1: Hansatech Instruments Ltd., Pentney, UK) was used to determine the chlorophyll content.

### 2.3. Soil Fertility Analysis

Soil samples were collected from the pots when the crop was harvested to determine the available nitrogen (N), phosphorous (P), and potassium (K) concentrations. Soil organic matter was determined using Walkley and Black’s [44] method. Extraction with sodium bicarbonate, alkaline potassium permanganate [45], and ammonium acetate [46] was carried out using standard protocols and procedures were applied to measure the available N, P, and K, respectively. Soil respiration (SR) was determined according to the procedure described by Dinesh et al. [47].

### 2.4. Data Interpretation

The collected data were analyzed using the computer software Statistix 8.1 at the 95% probability level. The different lowercase letters indicate the significant differences among various treatments.

## 3. Results

### 3.1. Wheat Growth Traits

The highest plant height was recorded in T_2_ (97.48 cm and 98.11 cm) followed by T_1_ (95.07 cm and 95.55 cm) and the lowest results were obtained in T_3_ (84.10 cm and 83.38 cm) (Table 2). The highest spike length was recorded in T_2_ (21.41 cm and 21.89 cm), followed by T_1_ (19.41 cm and 20.03 cm), and the lowest results were obtained in T_3_ (12.45 cm and 12.96 cm) (Table 2). Spikelets per spike were also significantly affected by all studied treatments. The highest number of spikelets per spike was recorded in T_2_ (30.19 and 30.65), in which control conditions were maintained and the seeds had been inoculated with plant-growth-promoting rhizobacteria (PGPR), followed by T_1_ (28.54 and 28.95), with control conditions and no PGPR inoculation, and the lowest results were obtained in T_3_ (20.22 and 20.73), in which deficit irrigation was applied without the use of PGPR (Table 2).

### 3.2. Wheat Yield Traits

The thousand-grain weight was significantly affected by PGRP inoculation and different irrigation systems (Table 3). The highest 1000-grain weight was recorded in T_2_ (44.77 g and 45.92 g) followed by T_1_ (42.78 g and 43.82 g). The lowest results were obtained in T_3_ (31.82 g and 32.90 g) (Table 3). The grain yield per plant was also significantly affected by PGRP inoculation and different irrigation systems (Table 3). The highest grain yield per plant was recorded in T_2_ (1.256 g and 1.393 g) followed by T_1_ (1.183 g and 1.316 g). The lowest results were obtained in T_3_ (0.693 g and 0.490 g).

### 3.3. Relative Growth Rate of Wheat

The crop growth rate (CGR) was significantly affected by PGPR inoculation and different irrigation methods in wheat in both the tillering and flag leaf stages (Table 4). The highest CGR was recorded in T_2_ (tillering stage: 2.29 g m^−2^ day^−1^ and 2.32 g m^−2^ day^−1^; flag leaf stage: 9.75 g m^−2^ day^−1^ and 9.71 g m^−2^ day^−1^) followed by T_1_ (tillering stage: 2.14 g m^−2^ day^−1^ and 2.17 g m^−2^ day^−1^; flag leaf stage: 9.19 g m^−2^ day^−1^ and 9.22 g m^−2^ day^−1^). The lowest results were obtained in T_3_ (tillering stage: 1.64 g m^−2^ day^−1^ and 1.13 g m^−2^ day^−1^; flag leaf stage: 7.09 g m^−2^ day^−1^ and 7.12 g m^−2^ day^−1^). The relative growth rate (RGR) was also significantly affected by PGPR inoculation and different irrigation methods in wheat in both the tillering and flag leaf stages (Table 5). The highest RGR was recorded in T_2_ (tillering stage: 0.143 g m^−2^ day^−1^ and 0.148 g m^−2^ day^−1^; flag leaf stage: 4.580 g m^−2^ day^−1^ and 4.610 g m^−2^ day^−1^) followed by T_1_ (tillering stage: 0.134 g m^−2^ day^−1^ and 0.139 g m^−2^ day^−1^; flag leaf stage: 4.236 g m^−2^ day^−1^ and 4.263 g m^−2^ day^−1^). The lowest results were obtained in T_3_ (tillering stage: 0.076 g m^−2^ day^−1^ and 0.070 g m^−2^ day^−1^; flag leaf stage: 3.543 g m^−2^ day^−1^ and 3.483 g m^−2^ day^−1^) (Table 5).

### 3.4. Gas Exchange Rate and Photosynthetic Capacity

The net assimilation rate (NAR) was significantly affected by PGPR inoculation and different irrigation methods in wheat in both the tillering and flag leaf stages (Table 6). The highest NAR was recorded in T_2_ (tillering stage: 1.530 g m^−2^ day^−1^ and 1.527 g m^−2^ day^−1^; flag leaf stage: 4.620 g m^−2^ day^−1^ and 4.643 g m^−2^ day^−1^) followed by T_1_ (tillering stage: 1.423 g m^−2^ day^−1^ and 1.431 g m^−2^ day^−1^; flag leaf stage: 4.433 g m^−2^ day^−1^ and 4.446 g m^−2^ day^−1^). The lowest results were obtained in T_3_ (tillering stage: 1.042 g m^−2^ day^−1^ and 1.020 g m^−2^ day^−1^; flag leaf stage: 3.633 g m^−2^ day^−1^ and 3.640 g m^−2^ day^−1^).

The leaf area index (LAI) was significantly affected by PGPR inoculation and different irrigation methods in wheat in both the tillering and flag leaf stages (Table 7). The highest LAI was recorded in T_2_ (tillering stage: 2.51 and 2.63; flag leaf stage: 7.35 and 7.43) followed by T_1_ (tillering stage: 2.32 and 2.41; flag leaf stage: 7.06 and 7.12) and the lowest results were obtained in T_3_ (tillering stage: 1.81 and 1.89; flag leaf stage: 6.01 and 6.14) in both years of the experiment.

**Figure 1:**
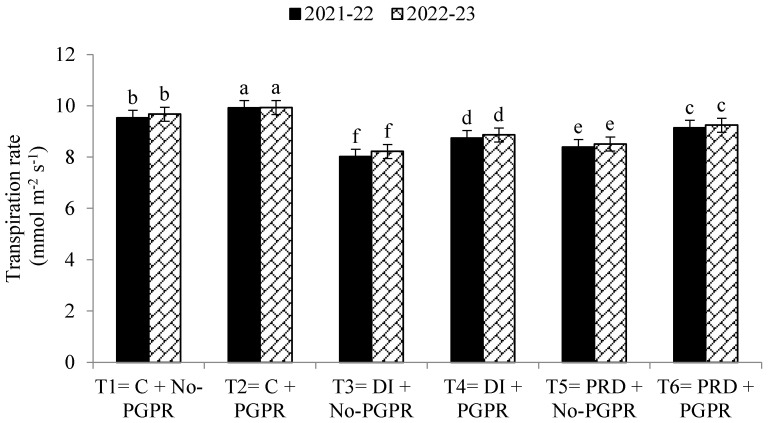
Effects of PGPR on the transpiration rate of wheat under different irrigation systems. The different letters show a significant difference in treatments at 5% probability level. PGPR (plant-growth-promoting rhizobacteria), C (control), DI (deficit irrigation), PRD (partial root drying).

**Figure 2:**
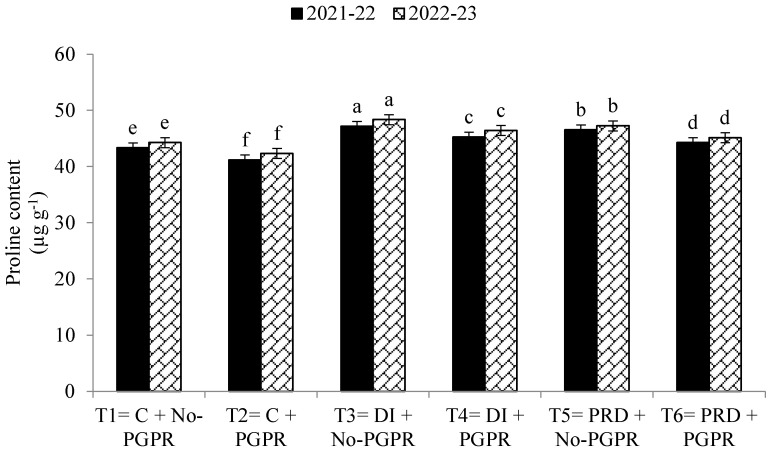
Effects of PGPR on the proline contents of wheat under different irrigation systems. The different letters show a significant difference in treatments at 5% probability level. PGPR (plant-growth-promoting rhizobacteria), C (control), DI (deficit irrigation), PRD (partial root drying).

**Figure 3:**
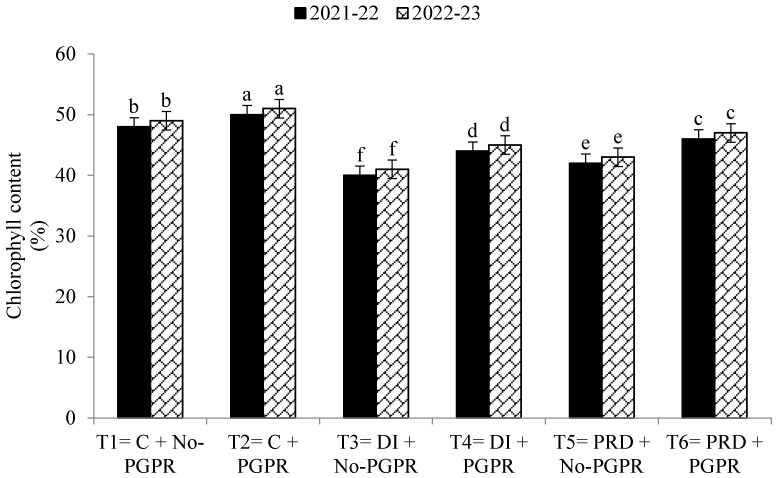
Effects of PGPR on the chlorophyll contents of wheat under different irrigation systems. The different letters show a significant difference in treatments at 5% probability level. PGPR (plant-growth-promoting rhizobacteria), C (control), DI (deficit irrigation), PRD (partial root drying).

**Figure 4:**
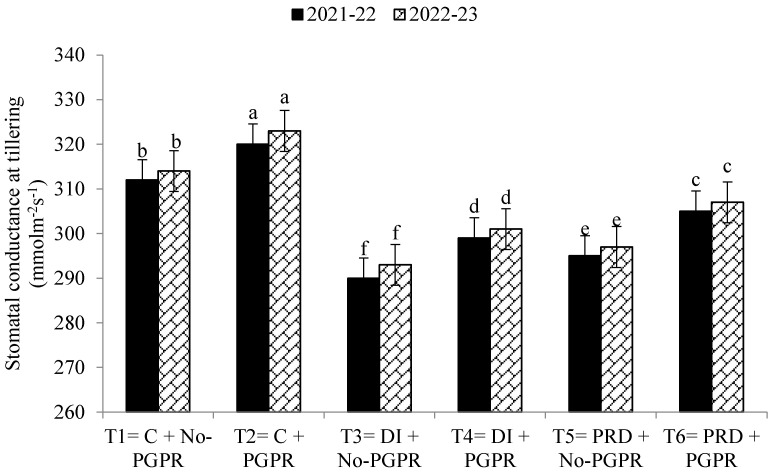
Effects of PGPR on the stomatal conductance at tillering stage of wheat under different irrigation systems. The different letters show a significant difference in treatments at 5% probability level. PGPR (plant-growth-promoting rhizobacteria), C (control), DI (deficit irrigation), PRD (partial root drying).

**Figure 5:**
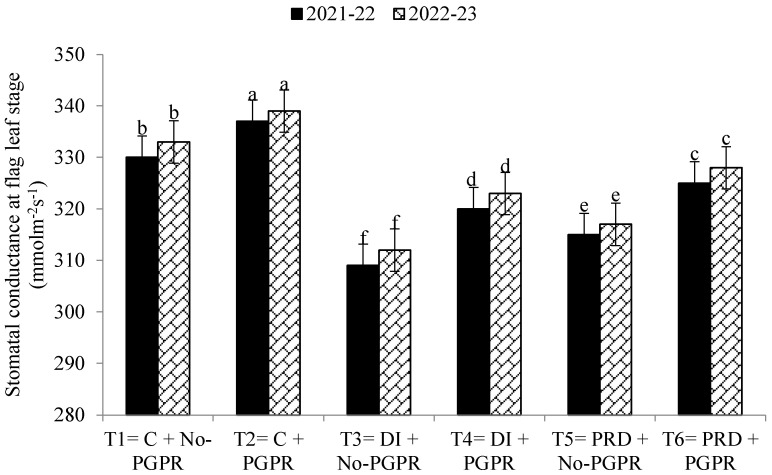
Effects of PGPR on the stomatal conductance at flag leaf stage of wheat under different irrigation systems. The different letters show a significant difference in treatments at 5% probability level. PGPR (plant-growth-promoting rhizobacteria), C (control), DI (deficit irrigation), PRD (partial root drying).

**Figure 6:**
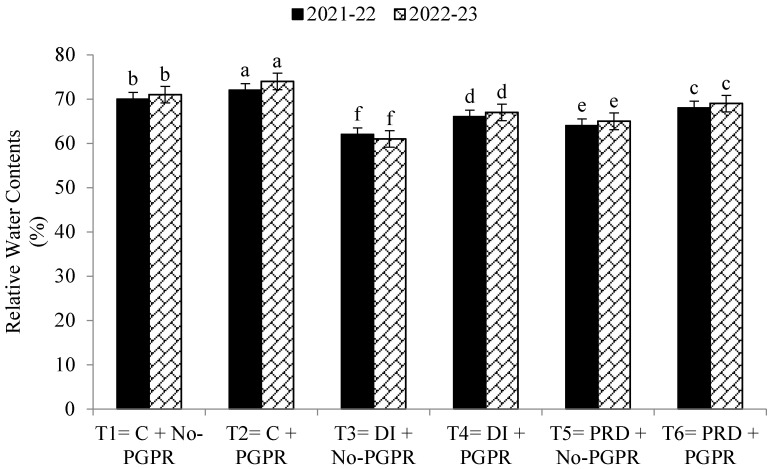
Effect of PGPR on the relative water contents (RWC) at flag leaf stage of wheat under different irrigation systems. The different letters show a significant difference in treatments at 5% probability level. PGPR (plant-growth-promoting rhizobacteria), C (control), DI (deficit irrigation), PRD (partial root drying).

Data regarding the transpiration rate, proline contents, chlorophyll contents, stomatal conductance, and relative water content were also significantly affected by the application of PGPR and by the different irrigation methods (Figure 1, Figure 2, Figure 3, Figure 4, Figure 5 and Figure 6). The highest values were recorded in T_2_, followed by T_1_, and the lowest results were noticed in T_3_ in both years.

### 3.5. Soil Fertility Indices

Soil properties such as available soil nitrogen, phosphorus, and potassium, the soil organic matter content, and soil respiration were significantly affected by all the treatments (Table 8). The highest soil N, P, and K concentrations, organic matter contents, and soil respiration were recorded in T_2_ followed by T_1_ and the lowest results were observed in T_3_. PRD irrigation resulted in higher values than DI irrigation, but we observed even higher values with PGPR inoculation.

## 4. Discussion

Availability of water is one of the serious challenges for the present and future of the world. Drought-dominant areas are increasing day by day in the world, which limits crop production [48]. One of the methods for water saving is deficit irrigation (DI) of the plants. It reduces the vegetative growth of plants and subsequently reduces their competitiveness with other plants and significantly reduces the cost of agricultural management practices [49]. However, partial root drying (PRD) is a changed type of deficit irrigation system [50]. In this method, during each irrigation, we apply irrigation to only half of the plant root, and in this way one part of the root absorbs water and the other remains dry for the next irrigation. Therefore, the partial root drying technique is considered an imperative irrigation strategy. Further, it has also been studied that exogenous application of PGRs like SA and PN along with medium levels of DI can help in rational utilization of water resources and could effectively improve crop yield under water-scarce conditions [51].

In the current research, plant height was significantly affected by the application of plant-growth-promoting rhizobacteria. The production of different growth hormones is also reported in the seed inoculation of wheat with *Azospirillum brasilense* [4]. Higher growth hormones can also improve plant growth through various physiological mechanisms [52]. Similar to our findings, Ahmad et al. [53] also reported that the application of PGPR produced more plant leaves for photosynthesis under water deficit conditions. A 5% greater plant height was also observed with the application of plant-growth-promoting rhizobacteria. The partial root-zone drying (PRD) system has been found to be more effective in increasing plant height than deficit irrigation. However, it was also stated that water deficit conditions also affected plant height [54], but the application of PGPR significantly enhanced plant height, as it increased the availability of nutrients for plant growth [4,24]. Previously, Stikić et al. [55] also reported greater plant height in PRD treatments as compared to deficit irrigation conditions [51]. In this study, the spike length, number of grains per spike, and grain weight of the wheat crop improved with the application of PGPR. This improvement might be due to the fact that PGPR produces the enzyme ACC deaminase, which helps to improve nutrient availability and reduce the extra ethylene, so there is greater photosynthetic production that improves the spike length in plants [4,24]. Water is essential to maintain all physiological functions of the plant, so water-deficient conditions severely reduce all the growth-related parameters of wheat plants [56]. Recently, Iqbal et al. [57] also proved that PRD irrigation is more beneficial to plant growth than the DI irrigation system. In PRD irrigation, more photosynthates are produced as compared to DI because of the plant’s stomatal size and internal signaling [58].

Water availability under PRD irrigation is more beneficial compared to DI, and the findings of the present study for the irrigation system are in line with those of Iqbal et al. [57] and Ahmad et al. [59]. The PRD irrigation system with the application of PGPR has been found to be more beneficial than the same system with non-inoculated wheat seeds [50]. Previously, Mayak et al. [60] reported that ACC enzyme production by PGPR-inoculated plants enhances nutrient uptake and increases the root and shoot length, which helps to improve plant growth- and yield-related parameters. Further, it was reported that PGPR inoculation in wheat seeds enhances plant growth by improving the organic entities for plant growth [61,62]. Recently, Zaheer et al. [63] also proved that application of PGPR produced more growth hormones such as cytokinin, which can improve cell division and, ultimately, growth- and yield-related parameters. The higher spike length, number of grains per spike, and 1000-grain weight directly help to increase the grain yield of the wheat crop [4]. It has been reported that PGPR produces the phytohormone indole-3-acetic acid (IAA), which can help to improve the surface area and adventitious root formation, thereby enhancing nutrient uptake and improving plant growth and yield [64,65]. Our study observed the same response with the PGPR-inoculated treatments in the FI, PRD, and DI irrigation systems, i.e., better plant growth and yields.

Previously, Bashan et al. [66] reported that inoculation of wheat seeds with A. brasilense enhanced photosynthesis, thus improving the plant growth rate and other physiological parameters. The application of PGPR also increased the leaf area index, and greater nutrient uptake was observed. Moreover, higher hormonal levels in crops have been observed after inoculation with PGPR, especially under water-limited conditions [14,67,68]. Ha et al. [69] also reported that cell division, nutrient uptake, and the leaf area index were higher following PGPR inoculation. In the PRD irrigation system, plant growth and yield were observed to be improved by PGPR inoculation in seeds. This study revealed that the transpiration rate in wheat plants was lower under DI and PRD irrigation systems compared to the control. However, PGPR significantly improved the transpiration rate with increased relative water content and stomatal conductance. Higher photosynthetic activities might be due to PGPR enhancing the production of cytokinin and other growth hormones, leading to improvements in plant growth, relative water content, and transpiration rate [5,70]. According to Iqbal et al. [14], water availability is essential for proper plant growth, but when water availability is restricted, the PRD irrigation system is more effective than the DI irrigation system [71].

Leaf area defines plants’ transpiration rate, stomatal conductance, and chlorophyll content, so a higher LAI leads to improved growth- and yield-related parameters. A higher LAI will be able to catch more solar energy for transpiration rate and photosynthesis [4,24,70]. Lazauskas et al. [72] reported that a lower LAI was observed under limited water availability, leading to poor leaf turgidity and leaf expansion. Concerning PRD and DI irrigation systems, the present findings are in line with those of Stikić et al. [55] and Elhani et al. [13]. Proline is also an important osmolyte in wheat plants, as it stabilizes the different organelles and molecules in the cell [73]. Szepesi and Szőllősi [74] reported lower proline contents in plants under limited water availability due to the breakdown of the protein molecule. Moreover, ABA regulates P5CS gene expression, increasing proline synthesis when water availability is lower [75]. Soil moisture is essential for the availability of soil nutrients. Higher concentrations of available soil N, P, and K were observed in the PRD irrigation system compared to the DI irrigation system. Iqbal et al. [14] reported that an appropriate soil moisture content is essential to enhance the availability of N, P, and K. PGPR fixes atmospheric nitrogen and enhances the availability of the nutrients by breaking down the complex molecules from the soil and enhancing the organic matter content [54,76]. Soil respiration is very suitable for plant growth and microorganisms, and it also indicates the soil’s microbial activity and organic matter content [63]. The present findings are aligned with those of Skopp et al. [76], who reported that limited soil moisture decreases microbial activity, organic matter content, and availability of soil nutrients. Greater microbial activity enhances soil respiration and organic matter, improving nutrient availability and source–sink linkage between soil nutrients [77].

Soil moisture affects the mineralization of soil nutrients. Under limited water conditions, more nitrogen is lost because of the increasing asynchronicity between nitrogen uptake and mineralization [78]. Hailegnaw et al. [79] and Elkhlifi et al. [80] reported that any soil amendment that can enhance the organic matter content of the soil could enhance soil nutrient availability. Further, application of PGPR has been shown to increase soil nutrients by breaking down the chelate compounds and increasing the amount of organic matter [63]. According to Al-Wabel et al. [81], PGPR enhances soil microbial activity, enhancing soil respiration. A higher organic matter content can also help to increase soil respiration. Limited water conditions decrease the soil’s microbial activity and organic matter content [14,63]. Our study also demonstrated that the PRD irrigation system with PGPR inoculation in seeds is more beneficial than the DI irrigation system with PGPR. The PRD irrigation system is more beneficial than the DI irrigation system, as soil nutrients are more available for plant uptake [14].

## 5. Conclusions

Plant-growth-promoting rhizobacteria (PGPR) improved wheat growth and physiology, particularly in conditions of water scarcity. Among the irrigation methods, partial root-zone Ddying (PRD) emerges as a promising approach compared to deficit irrigation (DI). PGPR inoculation showcases consistent efficacy within both PRD and DI irrigation systems, fostering improved wheat growth, yield characteristics, and soil fertility dynamics. The integration of PGPR inoculation with diverse irrigation strategies warrants further investigation for its synergistic benefits and optimal agricultural outcomes.

## Figures and Tables

**Table 1:** Properties of the soil used for the experiment.

Parameters	2021-22	2022-23
Organic matter (%)	0.98	0.71
pH	7.42	7.57
EC (µS/cm)	226	231
T.S.S. (%)	0.47	0.57
Available-P (ppm)	6.09	5.21
Available-P (ppm)	116	111
Saturation percentage	37	32
Soil separates		
Sand (%)	35	36
Sand (%)	41	39
Sand (%)	24	25
Texture of Soil	Loam Soil	Loam Soil

**Table 2:** Effects of plant-growth-promoting rhizobacteria on the plant height, spike length, and number of spikelets per spike of wheat under different irrigation systems.

Treatment	Plant Height (cm)		Spike Length (cm)		Number of Spikelets per Spike	
	2021-22	2022-23	2021-22	2022-23	2021-22	2022-23
T_1_ = Control + No PGPR	95.07 b	95.55 b	19.41 b	20.03 b	28.54 b	28.95 b
T_2_ = C + PGPR	97.48 a	98.11 a	21.41 a	21.89 a	30.19 a	30.65 a
T_3_ = DI + No PGPR	84.10 f	83.38 f	12.45 f	12.96 f	20.22 e	20.73 f
T_4_ = DI + PGPR	90.74 d	91.78 d	16.45 d	16.90 d	24.32 c	24.77 d
T_5_ = PRD + No PGPR	87.70 e	89.00 e	14.52 e	15.00 e	22.23 d	22.66 e
T_6_ = PRD + PGPR	92.78 c	93.48 c	17.11 c	17.53 c	26.23 c	26.70 c

The different letters show a significant difference in treatments at 5% probability level. PGPR (plant-growth-promoting rhizobacteria); C (control); DI (deficit irrigation); and PRD (partial root drying).

**Table 3:** Effects of PGPR on the thousand-grain weight and grain yield per plant of wheat under different irrigation systems.

Treatment	1000 GW		Yield per Plant	
	2021-22	2022-23	2021-22	2022-23
T_1_ = Control + No PGPR	42.78 b	43.81 b	1.183 b	1.316 b
T_2_ = C + PGPR	44.77 a	45.92 a	1.256 a	1.393 a
T_3_ = DI + No PGPR	31.82 f	32.90 f	0.693 f	0.490 f
T_4_ = DI + PGPR	37.66 d	38.84 d	0.913 d	0.890 d
T_5_ = PRD + No PGPR	34.32 e	35.25 e	0.816 e	0.753 e
T_6_ = PRD + PGPR	39.74 c	40.66 c	1.030 c	1.090 c

The different letters show a significant difference in treatments at 5% probability level. PGPR (plant-growth-promoting rhizobacteria); C (control); DI (deficit irrigation); and PRD (partial root drying).

**Table 4:** Effects of PGPR on the crop growth rate (CGR) of wheat under different irrigation systems.

Treatment	Crop Growth Rate at Tillering (g m^−2^ day^−1^)		Crop Growth Rate at the Flag Leaf Stage (g m^−2^ day^−1^)	
	2021-22	2022-23	2021-22	2022-23
T_1_ = Control + No PGPR	2.14 b	2.17 b	9.19 b	9.22 b
T_2_ = C + PGPR	2.29 a	2.32 a	9.75 a	9.71 a
T_3_ = DI + No PGPR	1.64 f	1.13 f	7.09 f	7.12 f
T_4_ = DI + PGPR	1.84 d	1.15 d	8.19 d	8.22 d
T_5_ = PRD + No PGPR	1.74 e	1.77 e	7.69 e	7.71 e
T_6_ = PRD + PGPR	1.94 c	1.97 c	8.69 c	8.72 c

The different letters show a significant difference in treatments at 5% probability level. PGPR (plant-growth-promoting rhizobacteria); C (control); DI (deficit irrigation); and PRD (partial root drying).

**Table 5:** Effects of PGPR on the relative growth rate (RGR) of wheat under different irrigation systems.

Treatment	Relative Growth Rate at Tillering (g m^−2^ day^−1^)		Relative Growth Rate at the Flag Leaf Stage (g m^−2^ day^−1^)	
	2021-22	2022-23	2021-22	2022-23
T_1_ = Control + No PGPR	0.134 b	0.139 b	4.236 b	4.263 b
T_2_ = C + PGPR	0.143 a	0.148 a	4.580 a	4.610 a
T_3_ = DI + No PGPR	0.076 f	0.074 f	3.543 f	3.483 f
T_4_ = DI + PGPR	0.104 d	0.108 d	3.856 d	3.826 d
T_5_ = PRD + No PGPR	0.086 e	0.085 e	3.676 e	3.680 e
T_6_ = PRD + PGPR	0.122 c	0.127 c	4.016 c	4.076 c

The different letters show a significant difference in treatments at 5% probability level. PGPR (plant-growth-promoting rhizobacteria); C (control); DI (deficit irrigation); and PRD (partial root drying).

**Table 6:** Effects of PGPR on the net assimilation rate (NAR) of wheat under different irrigation systems.

Treatments	NAR at Tillering (g m^−2^ day^−1^)		NAR at the Flag Leaf Stage (g m^−2^ day^−1^)	
	2021-22	2022-23	2021-22	2022-23
T_1_ = Control + No PGPR	1.423 b	1.431 b	4.433 b	4.446 b
T_2_ = C + PGPR	1.530 a	1.527 a	4.620 a	4.643 a
T_3_ = DI + No PGPR	1.042 f	1.020 f	3.633 f	3.640 f
T_4_ = DI + PGPR	1.234 d	1.224 d	4.023 d	4.036 d
T_5_ = PRD + No PGPR	1.125 e	1.129 e	3.830 e	3.846 e
T_6_ = PRD + PGPR	1.320 c	1.318 c	4.243 c	4.263 c

The different letters show a significant difference in treatments at 5% probability level. PGPR (plant-growth-promoting rhizobacteria); C (control); DI (deficit irrigation); and PRD (partial root drying).

**Table 7:** Effects of PGPR on the leaf area index (LAI) of wheat under different irrigation systems.

Treatment	Leaf Area Index at Tillering		Leaf Area Index at the Flag Leaf Stage	
	2021-22	2022-23	2021-22	2022-23
T_1_ = Control + No PGPR	2.32 b	2.41 b	7.06 b	7.12 b
T_2_ = C + PGPR	2.51 a	2.63 a	7.35 a	7.43 a
T_3_ = DI + No PGPR	1.70 f	1.83 f	6.01 f	6.14 f
T_4_ = DI + PGPR	1.98 d	1.99 d	6.57 d	6.65 d
T_5_ = PRD + No PGPR	1.81 e	1.89 e	6.31 e	6.47 e
T_6_ = PRD + PGPR	2.12 c	2.21 c	6.86 c	6.95 c

The different letters show a significant difference in treatments at 5% probability level. PGPR (plant-growth-promoting rhizobacteria); C (control); DI (deficit irrigation); and PRD (partial root drying).

**Table 8:** Effects of PGPR on the available soil N, P, and K, organic matter content, and soil respiration of wheat under different irrigation systems.

Treatment	Available Soil Nitrogen (mg kg^−1^)		Available Soil Phosphorus (mg kg^−1^)		Available Soil Potassium (mg kg^−1^)		Soil Organic Matter (%)		Soil Respiration (SR) (µg CO_2_-C g^−1^ day^−1^)	
	2021-22	2022-23	2021-22	2022-23	2021-22	2022-23	2021-22	2022-23	2021-22	2022-23
T_1_ = Control + No PGPR	30.14 b	29.15 b	14.30 b	13.21 b	60.37 b	58.26 b	1.31 b	1.12 b	24.11 b	25.21 b
T_2_ = C + PGPR	32.33 a	31.35 a	15.42 a	14.33 a	62.57 a	60.46 a	1.42 a	1.23 a	25.18 a	26.34 a
T_3_ = DI + No PGPR	22.21 f	21.23 f	10.42 f	09.33 f	52.45 f	50.34 f	0.98 f	0.71 f	20.14 f	21.34 f
T_4_ = DI + PGPR	26.32 d	25.36 d	12.31 d	11.22 d	56.58 d	54.47 d	1.17 d	0.94 d	22.16 d	23.34 d
T_5_ = PRD + No PGPR	24.12 e	23.15 e	11.41 e	10.32 e	54.37 e	52.26 e	1.09 e	0.83 e	21.47 e	22.31 e
T_6_ = PRD + PGPR	28.21 c	27.26 c	13.33 c	12.24 c	58.48 c	56.37 c	1.25 c	1.02 c	23.34 c	24.45 c

The different letters show a significant difference in treatments at 5% probability level. PGPR (plant-growth-promoting rhizobacteria), C (control), DI (deficit irrigation), PRD (partial root drying).

## Data Availability

The data is contained within the article.
